# Potential Pneumoconiosis Patients Monitoring and Warning System with Acoustic Signal †

**DOI:** 10.3390/s25061874

**Published:** 2025-03-18

**Authors:** Zhongxu Bao, Baoxuan Xu, Xuehan Zhang, Yuqing Yin, Xu Yang, Qiang Niu

**Affiliations:** 1 State Key Laboratory of Mining Response and Disaster Prevention and Control in Deep Coal Mines, Anhui University of Science and Technology, Huainan 232000, China; baozx@cumt.edu.cn; 2 School of Computer Science and Technology, China University of Mining and Technology, Xuzhou 221116, China; xubaoxuan@cumt.edu.cn (B.X.); xuehanzhang@cumt.edu.cn (X.Z.); yang_xu@cumt.edu.cn (X.Y.); niuq@cumt.edu.cn (Q.N.)

**Keywords:** inaudible acoustic sensing, pneumoconiosis, contactless sensing, commercial device

## Abstract

Monitoring for early symptoms is a critical step in preventing pneumoconiosis. The early signs of pneumoconiosis can be characterized by dyspnea, tachypnea, and cough. While traditional sensor-based methods are promising, they necessitate the wearing of devices and confine human physical movements. On the other hand, camera-based methods have issues related to illumination, obstruction, and privacy. Recently, wireless sensing has attracted a significant amount of research attention. Among wireless signals, acoustic signals possess unique advantages for fine-grained sensing due to their low propagation speed in the air and low hardware requirement. In this paper, we propose a system called $P^{3}Warning$ to realize low-cost warnings for potential pneumoconiosis patients in a contactless manner. For the first time, the designed system utilizes the inaudible acoustic signal to monitor early symptoms of pneumoconiosis (i.e., abnormal respiration and cough), leveraging a pair of commercial speaker and microphone. We introduce and address unique technical challenges, such as formulating a delay elimination method to synchronize transceiver signals and providing a search-based strategy to amplify signal variation for accurate and long-distance vital sign sensing. Ultimately, we apply an innovative signal decomposition technique to reconstruct the respiration waveform and extract features for cough detection. Comprehensive experiments were conducted to evaluate $P^{3}Warning$. Experiment results show that it can achieve a robust performance with a median error of 0.39 bpm for abnormal respiration pattern monitoring and an accuracy of 95% for cough detection in total, and support the furthest sensing range of up to 4 m.

## 1. Introduction

Pneumoconiosis is one of the most common occupational diseases, and 860,000 cases were reported in China by 2020 [1]. While various prevention methods have been implemented for many years, it is still a serious issue worldwide. Since pneumoconiosis is latent and incurable [2], it is crucial to monitor early symptoms, including abnormal respiration (i.e., dyspnea and tachypnea) and cough, to detect potential pneumoconiosis.

Traditional vital sign monitoring methods are based on wearable devices such as ECG [3,4], PSG [5,6], and accelerometers [7,8]. While promising in monitoring accuracy, wearable devices limit human physical activities and especially bring inconvenience to engineering operations. Among contactless vital sign monitoring methods, camera-based methods [9] have issues regarding lighting conditions, occlusion, and privacy. In recent years, wireless sensing has become a hot research area. Diverse wireless signals have been employed for vital sign monitoring, such as WiFi [10,11], RFID [12], radar [13], LoRa [14,15], and visible light [16,17]. Different from traditional sensor-based sensing, wireless sensing relies on analyzing the wireless signals reflected from the target to obtain vital sign information. However, several limitations are restricting the broad deployment of these methods. WiFi-based methods are seriously affected by environmental interference. Methods based on other wireless signals require dedicated high-cost hardware.

Acoustic signals [18,19] are mechanical wave vibrations propagating through a medium with basic characteristics such as frequency, amplitude, and phase that can be sensed by living beings or detected by sensors. Among wireless signals, acoustic signals have unique advantages: their low propagation speed (340 m/s) makes them suitable for fine-grained sensing, e.g., identifying breathing and coughing characteristics of human body through phase changes; meanwhile, loudspeakers and microphones are widely used in daily electronic devices, which provide a promising application for low-cost vital signs monitoring. With the characteristics of non-invasiveness, high sensitivity, and multi-dimensional information, acoustic signals show strong application potential and wide applicability.

In this paper, we propose a low-cost system called $P^{3}Warning$ for contactless monitoring of early pneumoconiosis symptoms, including abnormal breathing patterns and cough. $P^{3}Warning$ utilizes a pair of commercial speaker and microphone to transceiver inaudible acoustic signals [20] within the frequency range of 18 kHz∼22 kHz (audible sound is in 20 Hz∼16 kHz [21]). To achieve $P^{3}Warning$, we must tackle the following issues: (1) The transceiver signals lack clock synchronization, leading to a random delay in signal transmission by the speaker, which results in inaccurate target identification. (2) The sensing range is constrained, particularly in intricate environments. Acoustic signals experience significant propagation loss, leading to a reduced signal-to-noise ratio (SNR), and the signal variations from distant targets are prone to being obscured by background noise. (3) It is challenging to accurately isolate respiration and cough signals from the complex target signal.

To overcome the first issue, we suggest incorporating a preliminary acoustic signal to estimate the direct path, which can then be used to eliminate the random delay and synchronize the transceiver signals. For the second issue, we recommend a search-based approach to enhance the signal phase changes, facilitating the detection of targets at a greater distance. Lastly, we utilize an advanced version of ensemble empirical mode decomposition with adaptive noise (ICEEMDAN) for reconstructing irregular respiration patterns and apply feature extraction along with peak detection techniques for cough identification.

The main contributions of this work are listed as follows:

- To the best of our knowledge, it is the first time that commercial acoustic devices are applied to potential pneumoconiosis patients monitoring and warning via contactless sensing. We believe $P^{3}Warning$ is a critical step towards potential pneumoconiosis monitoring in the real world.
- We introduce an innovative approach for synchronizing acoustic signals by eliminating the unpredictable system latency from the speaker. Additionally, we advocate for a search-oriented technique to enhance signal phase differences, aimed at identifying targets at extended ranges. Finally, we propose a respiration extraction method by ICEEMDAN and extract suitable features to detect cough.
- We carry out extensive testing to assess the efficacy of $P^{3}Warning$. The findings indicate that $P^{3}Warning$ is capable of attaining a median error rate of 0.52 beats per minute (bpm) in monitoring irregular respiration patterns, with a 95% success rate in detecting coughs overall, and is effective up to a maximum distance of 4 m.

This paper is an extension of the proceedings paper [22]. The rest of this article is organized as follows. Section 2 introduces the related work of our research. Section 3 illustrates the fundamental principle of chirp-based acoustic sensing. Section 4 elaborates on the design of the $P^{3}Warning$ system. In Section 5, we conducted extensive experiments to evaluate the performance of $P^{3}Warning$. Finally, a brief conclusion is presented in Section 7.

## 2. Related Work

### 2.1. Vital Sign Monitoring

The current vital sign monitoring systems can be classified as device-based systems and device-free systems.

#### 2.1.1. Device-Based Vital Sign Monitoring

Device-based vital sign monitoring systems are typically based on medical sensors and smart wearable devices, which require users to wear devices on their bodies. Lazaro et al. [23] proposed a system for deriving respiratory rate from an armband, which records a three-channel electrocardiogram using three pairs of dry electrodes. ECG-derived respiration based on respiration-related modulation of QRS slopes and R-wave angle approach is used, and the respiratory rate from the armband obtains a relative error of 2.26% with respect to the ground truth. Shi et al. [24] proposed a wearable sensor based on fiber Bragg grating with a high sensitivity to achieve accurate and simultaneous measurement of respiration and heartbeat activities. The proposed sensor system can provide precise and clear waveforms of respiration and heartbeat. Qiu et al. [25] presented a wearable sensor patch with real-time respiration monitoring by measuring the change in thoracic impedance resulting from breathing. It can be used to monitor the respiratory rate accurately in static conditions with a low mean absolute error of 0.71 bpm. Jeon et al. [26] used the heart rate and 3-ACC signals measured on the smartwatch to predict sleep apnea in real time with an accuracy of 95%. These device-based systems limit human physical activities and bring inconvenience to life. Especially in the work environment, it is difficult for workers to accept wearing devices.

#### 2.1.2. Device-Free Vital Sign Monitoring

In contrast to device-based systems, device-free systems do not require direct contact between the users and the monitoring devices. Wireless sensing technologies have been widely employed for vital sign monitoring, including WiFi, radar, visible light, LoRa, and RFID. Wireless sensing relies on analyzing the wireless signals reflected from the target to obtain various vital signs. MoBreath [10] utilizes the WiFi channel state information readings extracted from the end-user device, a smartphone, to monitor the respiration rate. It can accurately estimate the respiration rate at a low error rate of 0.34 breaths per minute and support the sensing range of up to 3–4 m. Zhai et al. [27] proposed a method to measure respiratory motion with a single-chip millimeter-wave radar system. A simplified trunk model and a mode-decomposition-based respiration reconstruction are designed to analyze the 2D radar profile to extract non-stationary breathing motion information. Abuella et al. [16] proposed a contactless vital signs monitoring system that utilizes visible light sensing technology. The proposed system is implemented using a simple visible light source, photodetector, and data acquisition/processing unit. It is used with the developed signal processing algorithms to turn slight variations in reflected light power into accurate measurements of respiration and heart rate. Zhang et al. [28] sensed respiration by LoRa signals when the target was 25 m from the LoRa device. TagSleep [12] employs three RFID tags and one RFID reader to obtain two-layer information on sleep. The respiration sensing information is used as the basic first-layer information, which is applied to obtain further rich second-layer sensing information, including snore, cough, and somniloquy. Among these device-based systems, WiFi-based systems have a low sensing precision because of environmental interferences, and systems based on other signals usually require expensive dedicated devices.

### 2.2. Wireless Sensing Based on Acoustic Signals

In recent years, acoustic-based sensing has attracted extensive attention. Acoustic sensing technology utilizes acoustic signals to detect changes in angle, velocity, and phase, enabling fine-grained monitoring. It has been used in a number of applications, such as localization, gesture recognition, gait estimation, fall detection, eye blink detection, respiration monitoring, and heartbeat monitoring. RAILS [29] is an ultrasonic indoor localization system that can accurately and precisely locate a target in three dimensions using angle of arrival (AoA) measurements. The sensing range of the system can cover an area of 40 m^2^ with a maximum target–receiver distance of 10 m and an average standard deviation of 1 cm. Li et al. [30] enabled room-scale hand gesture recognition using increasingly popular smart speakers. They tested six commonly used hand gestures with an overall median gesture recognition accuracy of 97.25%. AcousticID [31] uses fine-grained gait information derived from acoustic signals generated by commercial off-the-shelf devices to identify human beings. It can identify different persons with an average accuracy of 96.6%. Lian et al. [32] developed a lightweight fall detection system by relying solely on a home audio device via inaudible acoustic sensing to recognize fall occurrences. It can achieve the precision and recall of 92.6% and 90.4%, respectively. BlinkListener [33] uses acoustic signals to sense the subtle eye blink motion by exploiting the harmful interference to maximize the subtle signal variation induced by eye blinks. It can achieve robust performance with a median detection accuracy of 95%. Wang et al. [34] designed and implemented a real-time and contactless respiration monitoring system by directly sensing the exhaled airflow from breathing using ultrasound signals. The system achieves a median error lower than 0.3 bpm for respiration monitoring and can accurately identify apnea. Zhang et al. [20] proposed a real-time heartbeat monitoring system, which employs a series of novel signal separation methods to extract the subtle heartbeat motion in the presence of strong interference from respiration. The system can achieve a median heart rate estimation error of 0.75 bpm and a median heartbeat interval estimation error of 13.28 ms. These acoustic-based works provide good basics for future research.

## 3. Preliminaries

**Figure 1:**
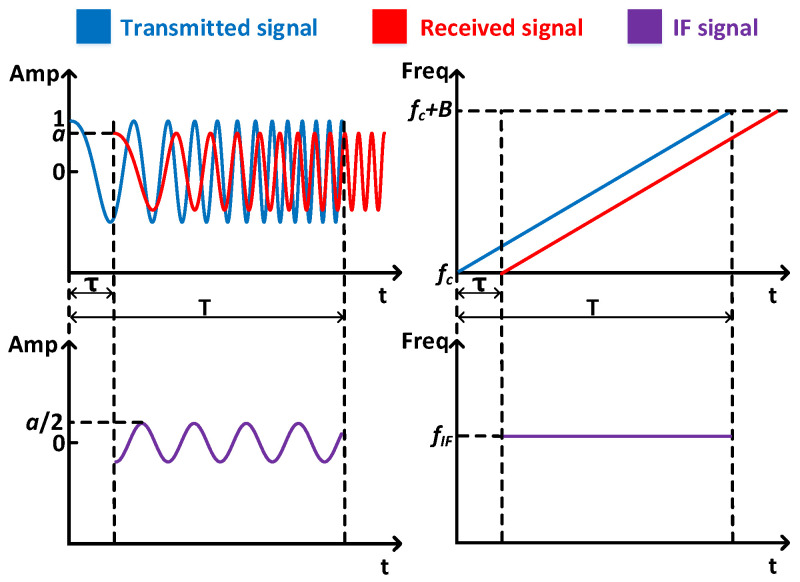
Acoustic sensing based on the chirp.

The chirp signal, frequently employed in acoustic sensing as referenced in [35], enables the differentiation of reflections originating from various distances. Figure 1 illustrates that a chirp signal is a sinusoidal wave with a frequency that increases linearly with time. The emitted signal can be expressed as

(1) $$S_{T}\left(t\right)=cos\left(\phi (t)\right)=cos\left(2\pi f_{c}t+\pi kt^{2}\right),$$

In this context, ϕ(t) denotes the instantaneous phase, $f_{c}$ represents the initial frequency, $k=\frac{B}{T}$ signifies the sweep rate, where *B* is the bandwidth of the frequency, and *T* denotes the duration of the chirp. The instantaneous frequency at any given time *t* is calculated as $\frac{1}{2\pi }\frac{d\left(\phi (t)\right)}{dt}=f_{c}+kt$, which is a linear function with respect to time. Once the transmitted signal bounces off the target, the received signal, which includes delay and attenuation, can be acquired and is represented as

(2) $$S_{R}\left(t\right)=acos\left(2\pi f_{c}\left(t-\tau \right)+\pi k{\left(t-\tau \right)}^{2}\right),$$

where *a* is the attenuation factor, $\tau =\frac{2R}{c}$ is the Time-of-Flight (ToF) of the signal, *R* is the distance from the target to the transceiver, and *c* is the acoustic signal propagation speed. The instantaneous frequency of the received signal is $f_{c}+k\left(t-\tau \right)$. The frequency difference between the transmitted and received signals can be represented as

(3) $$\left.f_{IF}={\left(f\right.}_{c}+kt\right)-\left(f_{c}+k\left(t-\tau \right)\right)=k\tau =k\frac{2R}{c}.$$

Therefore, the distance *R* from the target to the transceiver can be calculated by

(4) $$R=\frac{{cf}_{IF}}{2k}.$$

Next, we multiply the transmitted and received signals by the formula $cos\alpha \cdot cos\beta =\frac{1}{2}\left(\cos\left(\alpha +\beta \right)+\cos\left(\alpha -\beta \right)\right)$. And after using a low-pass filter, we can obtain the intermediate frequency (IF) signal, which can be represented as

(5) $$S_{IF}\left(t\right)=\frac{1}{2}acos\left(2\pi f_{IF}t+\phi_{c}\right),$$

where $\phi_{c}\approx 2\pi f_{c}\tau$ is the initial phase.

In practice, the transmitted signal is reflected by multipath. Therefore, the IF signal can be rewritten as a superposition of reflections from *N* paths,

(6) $$S_{IF}^{m}\left(t\right)=\frac{1}{2}\sum_{n=1}^{N}a_{n}\cos\left(2\pi f_{{IF}_{n}}t+\phi_{c_{n}}\right),$$

where $a_{n}$, $f_{{IF}_{n}}$ and $\phi_{c_{n}}$ are the attenuation factor, intermediate frequency, and initial phase of the $n^{th}$ path, respectively. As known in (5), signals reflected from objects at different distances lead to different intermediate frequencies. By performing the fast Fourier transform (FFT) on the IF signal, we can obtain multiple signals reflected from objects at different distances, and each signal represents a range bin. We can recognize the target by signal variations in range bins.

## 4. Architecture Design

### 4.1. Overview

**Figure 2:**
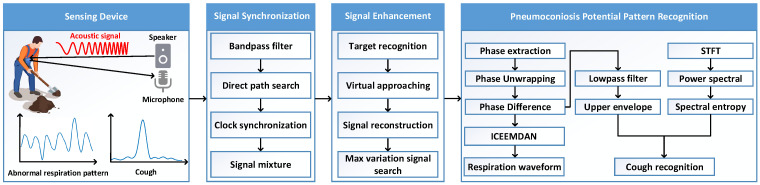
Overall architecture of $P^{3}Warning$, which shows sensing device, signal synchronization, signal enhancement, and pneumoconiosis potential pattern recognition.

Figure 2 shows the overall architecture of $P^{3}Warning$, including three main modules:

**Acoustic signal synchronization module.** We first filter out ambient noise in the received signal. Then, we make the received signal and the transmitted signal clock synchronized. Finally, we build the Intermediate Frequency (IF) signal whose frequency is the frequency difference of the received signal and the transmitted signal.

**Acoustic signal enhancement module.** We apply the virtual transceiver method to amplify the signal variation and thus increase the sensing range. And we propose a search-based strategy to extract the target-induced phase change.

**Pneumoconiosis potential pattern recognition module.** We achieve two functions, i.e., abnormal respiratory pattern monitoring and cough detection, and thus recognize early symptoms of pneumoconiosis. For abnormal respiration pattern monitoring, we utilize target-induced phase change to extract the target respiration waveform by ICEEMDAN. For cough detection, we extract two features, i.e., phase difference upper envelope and spectral entropy, and recognize cough signals by peak detection and threshold detection.

### 4.2. Acoustic Signal Synchronization

To ensure accurate transmission and reception of acoustic signals, the $P^{3}Warning$ system employs acoustic synchronization technology to eliminate delays caused by the lack of clock synchronization between the speaker and microphone. Using the cross-correlation method, the system calculates and removes the time delay in the signal. First, the system sends a known acoustic signal and synchronizes the speaker and microphone clocks by estimating the direct path. The cross-correlation analysis compares the waveforms of the transmitted and received signals, precisely determining the delay time, and then adjusts the received signal’s timing to synchronize it with the transmitted signal, ensuring the accuracy of the acoustic signals. This technology effectively enhances the precision of target recognition and signal extraction.

**Figure 3:**
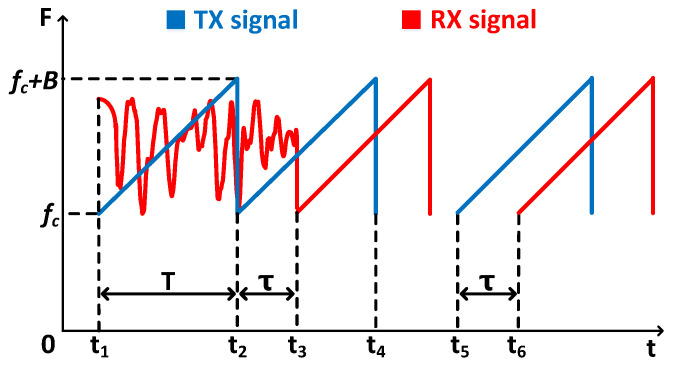
Acoustic signal synchronization.

**Figure 4:**
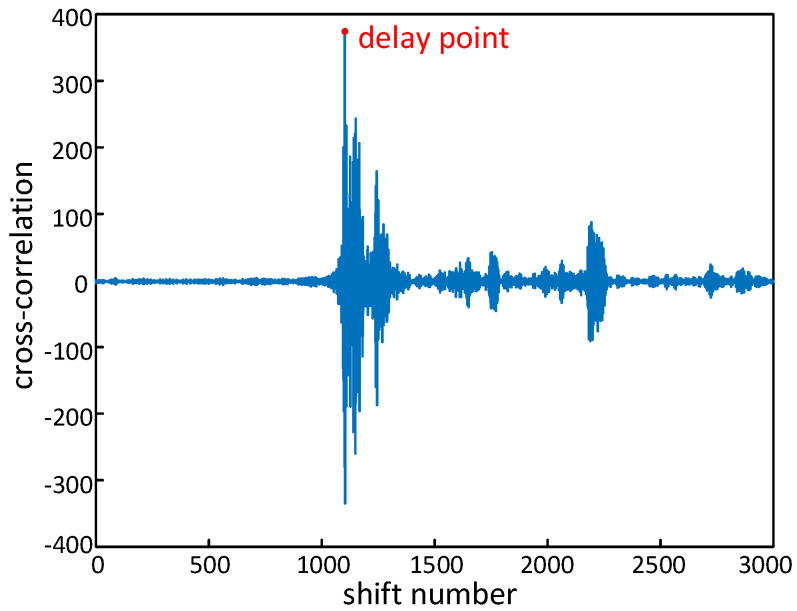
Cross−correlation of transmitted and received signals.

As introduced in Section 3, the IF signal utilizes ToF, i.e., delay between the transceiver signals, to sense targets at different distances, which relies on clock synchronization. However, there is a random transmitting time delay from the speaker, as the time $\left[0,\;t_{1}\right]$ in Figure 3. Ref. [36] exploits cross-correlation to find this time and clock synchronizes by subtracting this time delay in the received signal. The cross-correlation function between the transmitted signal $S_{T}\left(t\right)$ and received signal $S_{R}\left(t\right)$ is defined as

(7) $$R\left(\tau \right)=\left\{\begin{array}{c} \frac{1}{N-\tau }\sum_{n=0}^{N-Ø-1}\;S_{T}\left(n\right)\cdot S_{R}\left(n+\tau \right),\;\tau \geq 0\\ \frac{1}{N-|\tau |}\sum_{n=0}^{N-|\tau |-1}S_{R}\left(n\right)\cdot S_{T}\left(n+\tau \right),\;\tau <0 \end{array}\right.$$

where *N* is the number of samples in one chirp period *T*, and τ=−N+1,−N+2,…,N−1 is the shift number between $S_{T}\left(t\right)$ and $S_{R}\left(t\right)$. As shown in Figure 4, the random transmitting time delay $\hat{\tau }$ can be chosen by the maximum likelihood:

(8) $$\hat{\tau }=\underset{\tau }{\arg\;max}\left(R\left(\tau \right)\right).$$

However, the cross-correlation method is effective only when the time delay is less than one chirp period. If the time delay is larger than one chirp period, the cross-correlation function of signals in many periods has more than two peaks of the same size, so that we cannot choose the right delay.

After evaluating various speaker brands, we observed that there is frequently a time lag exceeding one chirp period, along with noise at a frequency close to the initial transmitted frequency, as depicted in Figure 3. To address these issues, we introduce an innovative signal transmission scheme. We initiate by broadcasting chirp signals in the interval $\left[0,\;t_{4}\right]$, followed by a silent signal in the interval $\left[t_{4},\;t_{5}\right]$, and conclude with continuous chirp signals for detection at time $t_{5}$. On the receiving side, we employ a bandpass filter [37] to eliminate low-frequency background noise. Subsequently, we identify the first instance post $t_{5}$ where the amplitude exceeds a predefined threshold (set at 0.001). This moment is approximated as the start of the direct path. We then isolate one chirp period of the received signal that includes this moment and conduct a cross-correlation with the transmitted signal over one chirp period. This process aligns the received signal with the transmitted signal in terms of clock synchronization. Ultimately, we combine the processed received signal with the transmitted signal to produce the intermediate frequency (IF) signal.

### 4.3. Acoustic Signal Enhancement

**Figure 5:**
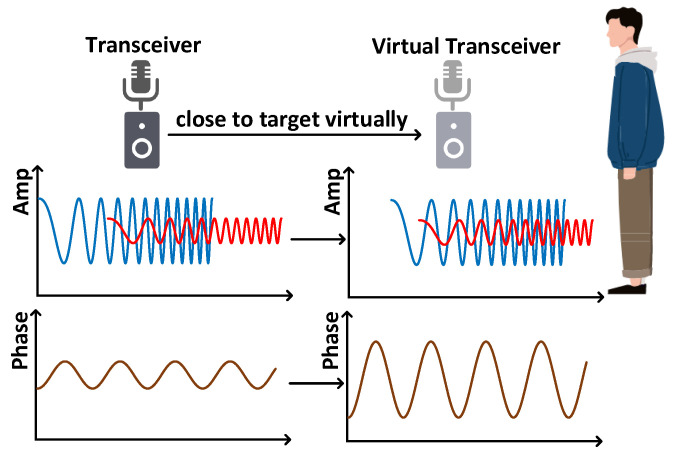
Virtual transceiver. (The blue waveform represents the reflected signal, the red waveform represents the original signal and the orange waveform represents the amplitude waveform.)

The reach of acoustic signals is constrained due to significant signal loss. Additionally, as the target recedes from the transceiver, the Time of Flight (ToF) for the signal increases, reducing the number of samples in the Intermediate Frequency (IF) signal and diminishing the phase shift caused by the target. Fortunately, we can enhance the IF signal’s sample count by employing the virtual transceiver technique [38], as illustrated in Figure 5. Essentially, we introduce a delay in the transmission signal prior to signal mixing, which effectively shortens the ToF. This virtual reduction in target–transceiver distance results in a higher sample count for the IF signal, thereby amplifying the phase variation and extending the sensing range.

To enhance signals more effectively, we first perform FFT on the IF signal and then find the range bin with the largest variance of signal variations, i.e., the range bin where the target locates. According to [38], the variance in the signal variation in the range bin where the target locates increases first. And then, the variance levels turn off when the target–transceiver distance is within 1 m. Therefore, we propose a search-based strategy to recognize the target. First, the virtual transceiver is moved directly to a distance of 1 m from the target. Then, we vary the distance between the target and the virtual transceiver from 1 m to 0.1 m at a step size of 0.1 m. At each step, a delay is calculated by the equation $∆\tau =\frac{2∆R}{c}$ and added to the transmitted signal. Finally, we reconstruct the IF signal and choose the signal with the largest variance as the final signal for extracting fine-grained activity information.

### 4.4. Pneumoconiosis Potential Pattern Recognition

#### 4.4.1. Fine-Grained Activity Information Extraction

As introduced in Section 3, we recognize the target bin because of its large signal variation. The initial phase of the signal in the target bin can be represented as $\phi_{c}=2\pi f_{c}\tau =\frac{4\pi f_{c}R}{c}$. Therefore, the phase change can be calculated by

(9) $$∆\phi =\frac{4\pi f_{c}∆R}{c},$$

where ∆R is the target displacement. If the starting frequency $f_{c}$ = 18 kHz, the phase change is 37.8° when the target moves 1 mm. This is enough for us to extract the target’s respiration and cough by their frequency features.

#### 4.4.2. Abnormal Respiration Pattern Monitoring

The ordinary respiration signals have a periodic change in the frequency range of 0.1 Hz∼0.5 Hz. But for the early pneumoconiosis, abnormal respiration patterns mainly contain dyspnea and tachypnea, which cause a bad periodicity of the respiratory waveform. As a result, we cannot adopt a simple filter to extract respiratory signals. Empirical mode decomposition (EMD) [39] is a signal time-frequency analysis algorithm, which decomposes a signal into a series of intrinsic mode functions (IMFs) with independent frequency. Every IMF satisfies two conditions: (a) The number of extrema and that of cross-zero points are extremely the same, or the number difference is at most one. (b) The mean value of the upper envelope formed by the local maxima and the lower envelope formed by the local minima is zero. The EMD process for the phase change in all sensing time ϕ(t) is as follows:

Step1: We identify all local extrema including maxima and minima of the phase change ϕ(t).

Step2: A cubic spline line is applied to connect the local maxima as the upper envelope $\phi_{u}\left(t\right)$ and connect the local minima to produce the lower envelope $\phi_{l}\left(t\right)$.

Step3: The mean of the upper and lower envelope is calculated by

(10) $$\phi_{m}\left(t\right)=\frac{1}{2}\left(\phi_{u}\left(t\right)-\phi_{l}\left(t\right)\right).$$

Step4: Calculate the intermediate component $m\left(t\right)=\phi \left(t\right)-\phi_{m}\left(t\right)$. If m(t) satisfies the conditions of IMF, it can be considered as the first IMF component denoted as $c_{1}\left(t\right)$. If not, repeat the above steps to m(t) until it satisfies the conditions of IMF.

Step5: Consider $\phi^{\prime }\left(t\right)=\phi \left(t\right)-c_{1}\left(t\right)$ as a new input and repeat steps 1–4 to obtain the second IMF component. The process is repeated until the decomposition is complete.

In this way, the phase change ϕ(t) is decomposed into a series of IMFs. The IMF in the frequency range of respiration can be sifted as the target respiration signal. However, the fast-changing local extreme values of the abnormal respiration may cause the mode mixing which leads to multiple frequency components in one IMF or one frequency component in multiple IMF components. Zhang et al. [20] adopt complete ensemble empirical mode decomposition with adaptive noise (CEEMDAN) to address this issue, but there are still residual noises and pseudo modes in sifted IMFs. Therefore, we propose to employ ICEEMDAN to obtain abnormal respiration waveforms. ICEEMDAN is different from CEEMDAN which directly adds Gaussian white noise in the decomposition process, but adds the $k^{th}$ IMF of the white noise decomposed by EMD.

Define that the phase change ϕ(t) is the original series, $w^{i}$ is the Gaussian white noise, $E_{k}\left(\cdot \right)$ is the $k^{th}$ IMF component decomposed by EMD, $\beta_{k}$ is the ratio of the added noise’s SNR to the noise component’s standard deviation, N(·) is the operator which calculates the local mean of the signal, and $\langle \cdot \rangle$ is the operator of averaging. The process of ICEEMDAN to obtain the respiration signal is as follows:

Step1: Add the white noise to the original series to construct a new sequence, which can be represented as

(11) $$\phi^{i}\left(t\right)=\phi \left(t\right)+\beta_{0}E_{1}\left(w^{i}\right).$$

Step2: By averaging the local mean of the new sequence $\phi^{i}\left(t\right)$, the first residual can be calculated by

(12) $$r_{1}=\langle N\left(\phi^{i}\left(t\right)\right)\rangle .$$

Step3: The first IMF can be calculated by

(13) $$c_{1}=\phi \left(t\right)-r_{1}.$$

Step4: Continue to add the white noise and use averaging the local mean to obtain the second residual, which can be calculated by

(14) $$r_{2}=\langle N\left(r_{1}+\beta_{1}E_{2}\left(w^{i}\right)\right)\rangle .$$

And the second IMF can be calculated by

(15) $$c_{2}=r_{1}-r_{2}=\langle N\left(\phi^{i}\left(t\right)\right)\rangle -\langle N\left(r_{1}+\beta_{1}E_{2}\left(w^{i}\right)\right)\rangle .$$

Step5: In the same way, the $k^{th}$ residual can calculated by

(16) $$r_{k}=\langle N\left(r_{k-1}+\beta_{k-1}E_{k}\left(w^{i}\right)\right)\rangle ,$$

and the $k^{th}$ IMF can be calculated by

(17) $$c_{k}=r_{k-1}-r_{k}.$$

Until the decomposition is complete, we can obtain all IMFs.

**Figure 6:**
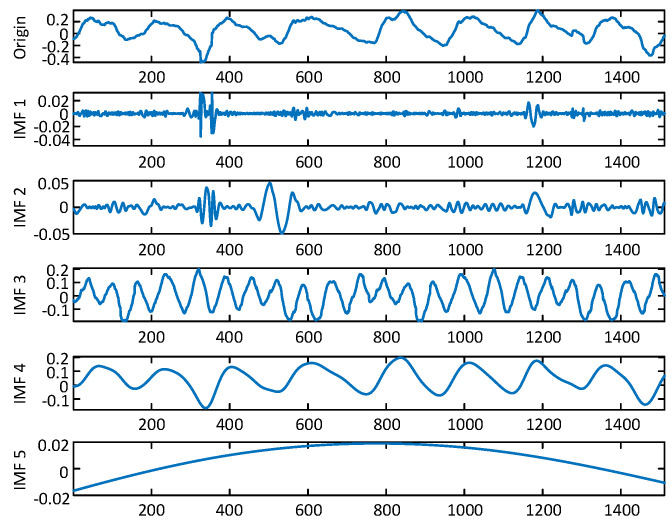
The phase change is decomposed by ICEEMDAN.

**Figure 7:**
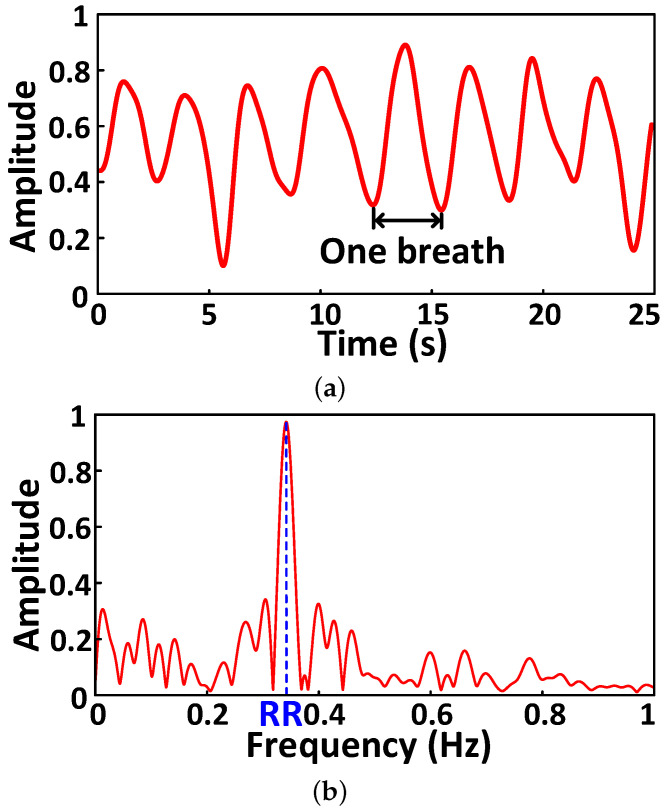
Abnormal respiration pattern monitoring. (**a**) Respiration waveform. (**b**) Respiration rate estimation.

As shown in Figure 6, the phase change is decomposed into five IMFs by ICEEMDAN. By converting each IMF into its frequency-domain signal, we can search for the signal in the frequency range of about 0.1 Hz∼0.5 Hz to obtain the target respiration signal, such as the IMF_4_. We can further perform FFT spectral estimation on the respiration signal to obtain the respiratory rate (RR) based on the peak size and harmonic characteristic, as shown in Figure 7. Therefore, we can monitor the abnormal respiration pattern, i.e., dyspnea and tachypnea.

#### 4.4.3. Cough Detection

Cough-induced phase changes in the elicited signals are easily masked by other fine-grained activities at similar frequencies, i.e., chest displacements cause rapid changes in air flow, which in turn cause phase changes in the high-frequency acoustic signals. However, other fine-grained activities, such as breathing or slight head and body movements, can also cause similar phase changes in a similar frequency range. This makes it difficult to distinguish the phase changes induced by coughing from the signal changes of other fine-grained activities. In order to effectively differentiate the cough signal from these similar frequency activities, the system uses feature extraction methods such as the upper envelope of the phase change and spectral entropy.

**Figure 8:**
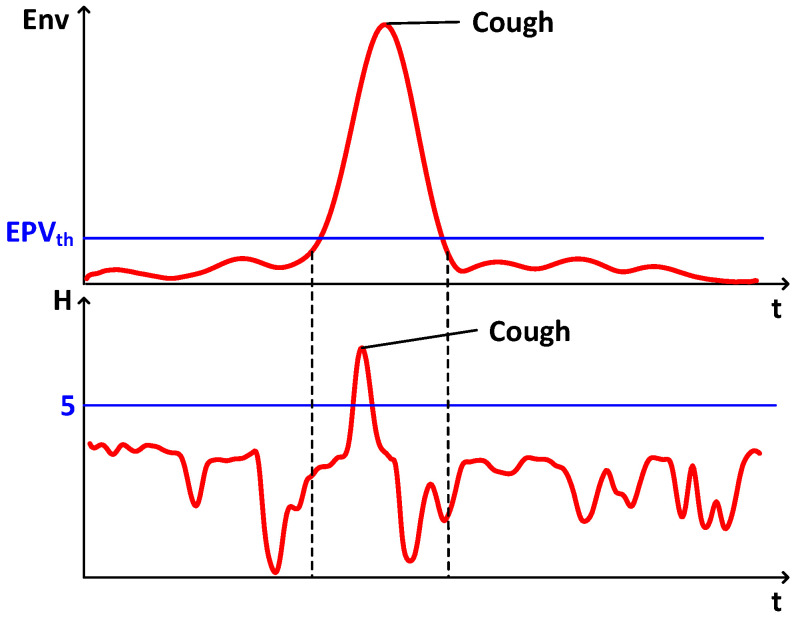
Cough detection.

The upper envelope of phase change highlights the fast and stronger phase changes caused by coughing by capturing the extremes of the signal amplitude changes, and the frequency and energy of the signal changes caused by coughing is slightly higher than that of breathing due to the larger and faster thoracic displacement caused by coughing. Therefore, we can extract the envelope over the phase change as a feature. The envelope is a curve that reflects the change in amplitude of the high frequency signal and can be expressed as

(18) $$Env\left(t\right)=\left|\phi \left(t\right)+j\cdot Hilbert\left(\phi (t)\right)\right|,$$

where $Hilbert\left(\phi (t)\right)$ is the Hilbert transform of ϕ(t), and ϕ(t) is the signal phase change after being filtered. As shown in the above waveform in Figure 8, the curve shows a clear peak when the target coughs.

The second feature is the spectral entropy. It describes the randomness of the energy distribution on the spectrogram. Cough signals are usually more sudden and random than other fine-grained activities, so cough signals usually have higher entropy than other fine-grained activities, as shown in the below waveform in Figure 8. To obtain the spectral entropy, we first perform the Short-Time Fourier Transform (STFT) on the received signal to obtain its spectrogram $S\left(f,t\right)$. Then, we calculate the power spectral density that can be expressed as

(19) $$P\left(f,t\right)=\frac{1}{f_{max}-f_{min}}\sum_{f=f_{min}}^{f_{max}}{\left|S\left(f,t\right)\right|}^{2},$$

where $f_{min}∼f_{max}$ is the frequency range of the spectrum. The power spectral density is then normalized to $p\left(f,t\right)$. Finally, the spectral entropy can be calculated by

(20) $$H\left(t\right)=-\sum_{f=f_{min}}^{f_{max}}p\left(f,t\right)\cdot \ln p\left(f,t\right).$$

Next, we utilize these two features to recognize the cough signal. We first recognize all peaks of the phase-change upper envelope, noted as $EPV=\left\{{epv}_{1}\ldots {epv}_{i}\ldots {epv}_{n}\right\}$. And the trough index pairs corresponding to peaks are noted as $ETI=\left\{\left\{{eti}_{11}\;{eti}_{12}\right\}\ldots \left\{{eti}_{i1}\;{eti}_{i2}\right\}\ldots \left\{{eti}_{n1}\;{eti}_{n2}\right\}\right\}$. We define a threshold as

(21) $${EPV}_{th}=avg\left(EPV\right)+k\cdot var\left(EPV\right),$$

where $avg\left(EPV\right)$ is the average of EPV, $var\left(EPV\right)$ is the variance of EPV, and the coefficient *k* is used to adjust the threshold appropriately according to the environment. Then, we record the trough index pairs corresponding to peaks larger than ${EPV}_{th}$ in EPV. For spectral entropy, we recognize the values and indices of all peaks. Then, we set a threshold (empirically set it as 5) and look for the index of all peaks larger than the threshold. If the found index is within the ranges of the recorded trough index pairs, we consider that the target coughs.

#### 4.4.4. Alarm

Finally, $P^{3}Warning$ monitors the target for a long time. If the target frequently have the symptoms of the abnormal respiration pattern (i.e., dyspnea and tachypnea) and cough, we will use the speaker to raise an alarm. $P^{3}Warning$ aims to monitor early pneumoconiosis symptoms for high-incidence population, such as coal miners. The $P^{3}Warning$ system pseudo-code flow is shown in Algorithm 1. If the high-incidence population of pneumoconiosis applies $P^{3}Warning$ and receive alarms, they can go to the hospital to further check whether suffering from pneumoconiosis.
**Algorithm 1:**$\;P^{3}Warning$ System Architecture Design
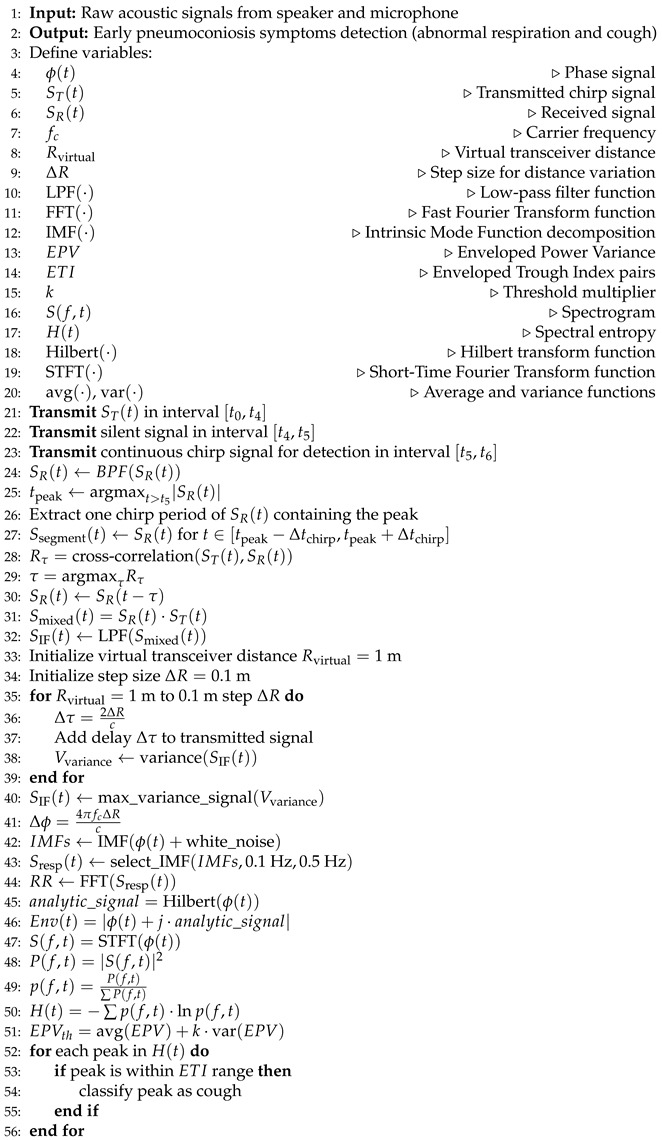

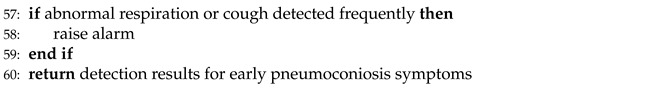


## 5. Evaluation

### 5.1. Experiment Setup

**Figure 9:**
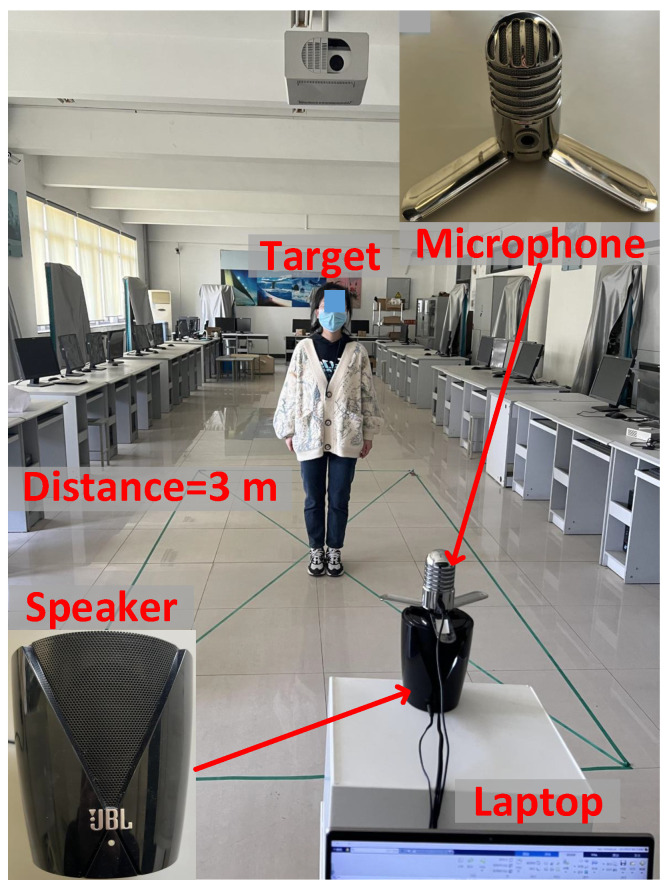
Experiment scenario and setup in a indoor laboratory.

As shown in Figure 9, we adopt a commercial speaker (JBL Jembe, 6 Watt, 80 dB) to transmit acoustic chirp signals and a commercial microphone (SAMSON MeteorMic, 16 bit, 48 kHz) as a receiver. We control the laptop (Dell Inspiron 7566) to transmit and receive acoustic signals and process the signals with MATLAB R2023a.For the acoustic signal transmitted by the speaker, the starting frequency $f_{c}$ = 18 kHz, the frequency bandwidth *B* = 4 kHz, and the chirp period *T* = 0.04 s. And the sampling rate of the microphone is 48 kHz.

We recruited 10 graduate volunteers to participate in the study, including two females and eight males.The two female volunteers are aged 24 and 26, all of whom are healthy with no chronic diseases or respiratory issues, have a normal weight, and some have mild allergic reactions. The eight male volunteers are aged 22, 29, 33, 27, 25, 30, 32, and 24, and their health status is also good, with no chronic diseases. Some have occasional seasonal allergies or mild shortness of breath. All male volunteers are physically fit, with some regularly exercising and maintaining a healthy lifestyle.

The experiments were conducted in a indoor laboratory and a coal mine laboratory. We collected breathing data of each volunteer for three hours in the indoor laboratory and one hour in the coal mine laboratory. The ground-truth respiration was obtained with a smart bracelet. And during the collection, they stood in front of the transceiver and engaged in three behaviors randomly, including talking, yawning, and coughing. In the evaluation, we evaluate the performance of the respiration monitoring and cough detection to validate our $P^{3}Warning$.

### 5.2. Performance Metrics

To evaluate the performance of our proposed method, we employ the respiration rate median error for the respiration monitoring, and the accuracy, precision, recall, and F1-score for the cough detection. Specifically, the four metrics of the cough detection can calculated by

(22) $$Accuracy=\frac{TP+TN}{TP+TN+FP+FN\prime }$$

(23) $$Precision=\frac{TP}{TP+FP\prime }$$

(24) $$Recall=\frac{TP}{TP+FN\prime }$$

(25) $$F1\;score=\frac{2\cdot Precision\cdot Recall}{Precision+Recall\prime }$$

where the true positive (TP) means that the cough is correctly detected, the true negative (TN) means that the non-cough is correctly detected, the false negative (FN) means missing alarm of cough detection, and the false positive (FP) means false alarm of cough detection.

### 5.3. Experiments in a Indoor Laboratory

#### 5.3.1. Overall Performance

**Figure 10:**
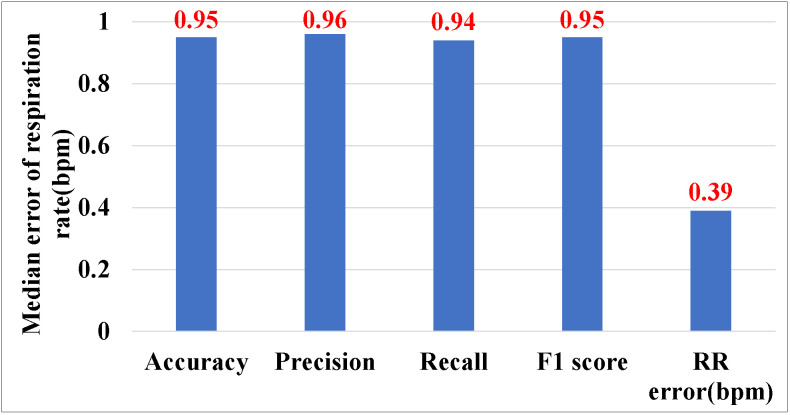
Overall performance of $P^{3}Warning$.

We first evaluate the overall performance of $P^{3}Warning$. As shown in Figure 10, $P^{3}Warning$ can achieve an excellent performance with a respiratory rate median error of 0.39 bpm, and accuracy, precision, recall and F1-score of 95%, 96%, 94%, and 95% for the cough detection, respectively. The results demonstrate $P^{3}Warning$’s effectiveness and robustness for the early symptom monitoring of pneumoconiosis.

#### 5.3.2. Evaluation of Acoustic Signal Synchronization

**Figure 11:**
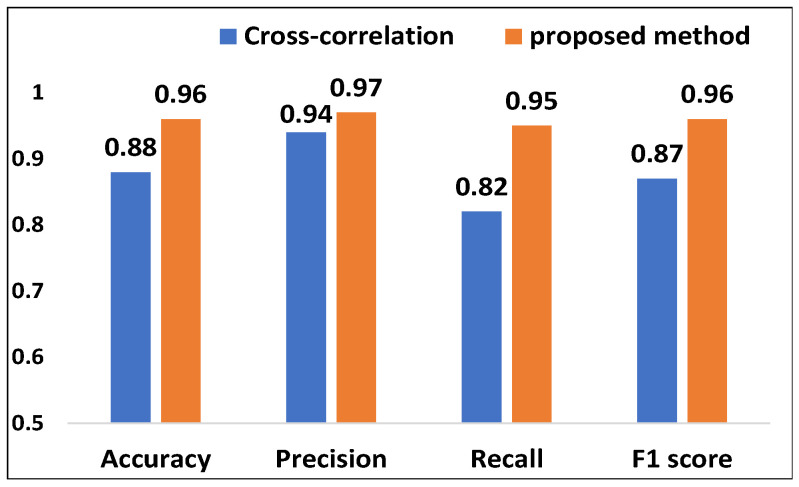
Evaluation of acoustic signal synchronization.

We demonstrate the effectiveness of our proposed synchronization method by comparing the performance impact of the cross-correlation [36] method in previous work with our method. Specifically, we asked the volunteer to experiment with a distance of 2 m from the transceiver. For abnormal respiration, with and without our proposed method, the respiratory rate median error is 0.31 bpm and 1.68 bpm, respectively. The results of cough detection are shown in Figure 11. When adopting our proposed method, the accuracy, precision, recall and F1-score are 96%, 97%, 95% and 96%, respectively. This is much higher than the performance when adopting the cross-correlation method. The results demonstrate that our proposed method can effectively solve the problem of acoustic signal synchronization.

#### 5.3.3. Evaluation of Acoustic Signal Enhancement

**Figure 12:**
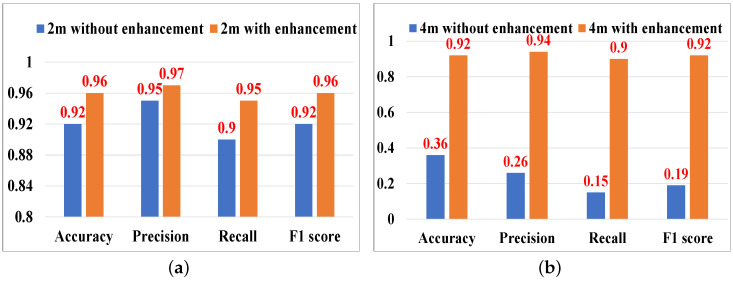
Evaluation of acoustic signal enhancement. (**a**) At 2 m. (**b**) At 4 m.

We adopt the virtual transceiver [38] method and a search-based strategy to increase the signal phase variation, thus increasing the sensing range of the acoustic signal in Section 4.3. To verify the method’s effectiveness, we evaluate the performance at a distance of 2 m and 4 m from the transceiver. For abnormal respiration, with and without our proposed method, the respiratory rate median error is 0.31 bpm and 1.81 bpm, respectively, at 2 m, and 0.56 bpm, and 5.75 bpm, respectively, at 4 m. The results of cough detection are shown in Figure 12. At 2 m, there is not much difference in the performance between the two cases. When the distance is 4 m, it is almost impossible to recognize respiration patterns and cough without our method, while high performance can still be achieved with the acoustic signal enhancement method. The experiment results show the effectiveness of the acoustic signal enhancement method for increasing the sensing range.

#### 5.3.4. Evaluation of Cough Feature Extraction

**Figure 13:**
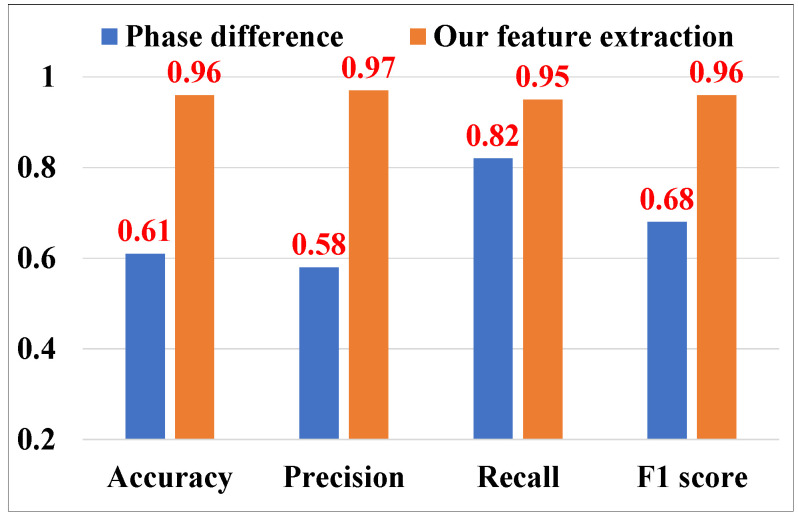
Evaluation of cough feature extraction.

To discern cough-induced signals amidst other subtle activities, we focus on two key features: the phase-difference upper envelope and spectral entropy, for cough detection. We tested the efficacy of these features through rigorous bench-marking. Volunteers were instructed to perform three distinct actions—speaking, yawning, and coughing—while standing 2 m away from the transceiver. We then assessed the system’s performance based on signal phase difference and feature extraction methods. The outcomes, depicted in Figure 13, indicate a marked improvement in performance with feature extraction. These findings confirm that our selected features can effectively distinguish coughs from other nuanced activities, thereby validating the enhancement in cough detection accuracy achieved through feature extraction.

#### 5.3.5. Impact of Different Distances

**Figure 14:**
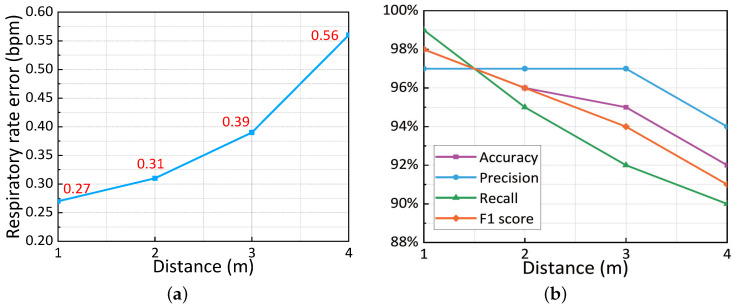
Performance of $P^{3}Warning$ at different distances. (**a**) Respiration monitoring. (**b**) Cough detection.

To assess the efficacy of $P^{3}Warning$ across various distances, we had a participant stand facing the transceiver, adjusting their position from 1 m to 4 m in 1-m increments. Figure 14 illustrates $P^{3}Warning$’s performance at these distances. At the closest range of 1 m, $P^{3}Warning$ delivers top-tier results, with a median respiratory rate error of 0.27 beats per min (bpm) and high metrics for cough detection: 98% accuracy, 97% precision, 99% recall, and 98% F1-score. As distance grows, performance declines slightly due to increased signal attenuation, which reduces the signal-to-noise ratio (SNR) and phase changes. However, even at the maximum distance of 4 m, $P^{3}Warning$ maintains respectable performance, with a median respiratory rate error of 0.56 bpm and cough detection metrics of 92% accuracy, 94% precision, 90% recall, and 91% F1-score. These findings confirm that $P^{3}Warning$ meets the needs for the majority of practical applications.

#### 5.3.6. Impact of Different Angles

**Figure 15:**
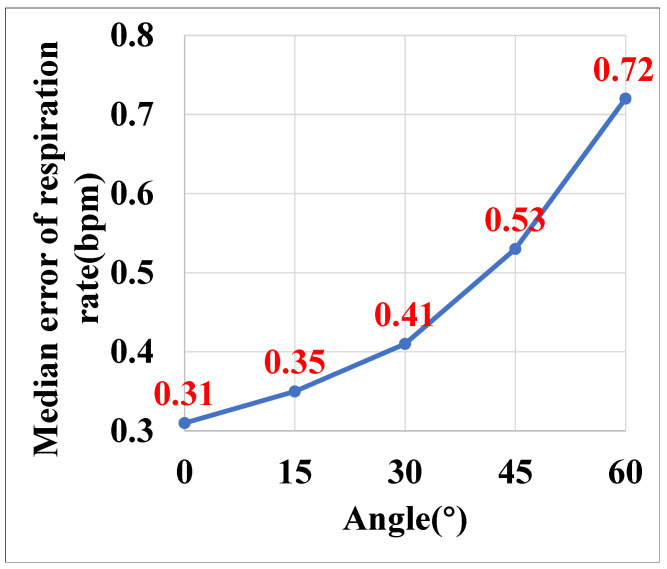
Positional errors in $P^{3}Warning$ respiration rates at different angles.

To evaluate the impact of the different target–transceiver angle, we asked the volunteer to stand at 2 m in front of the transceiver and varied the the angle between the volunteer and transceiver from 0° to 60° at a step size of 15°. The experimental results are shown in Figure 15. When the target–transceiver angle is 0°, $P^{3}Warning$ achieves the highest performance with a respiratory rate median error of 0.31 bpm, and accuracy, precision, recall, and F1-score of 96%, 97%, 95%, and 96%, respectively, for the cough detection. As the angle increases from 0° to 60°, the performance slightly decreases due to the high radiation directivity of high-frequency acoustic signals from commodity speakers. When the target–transceiver angle is over 60°, it is difficult to recognize the target with our method. The results demonstrate that $P^{3}Warning$ can work efficiently when the user is in different angles from 0°to 60° with respect to the transceiver.

At the same time, considering that the microphone is fixed to the receiver, the angle between the volunteer and the receiver is changing at the same time as the angle between the microphone and the target is changing, i.e., the microphone directionality between 0° and 60° has very little effect on the system performance.

#### 5.3.7. Impact of Different Microphones

The SAMSON Meteor Mic performs excellently in receiving high-frequency signals, especially within the target–receiver angle range of 0° to 60°. The system achieves a median respiratory rate error of 0.18 bpm and a cough detection accuracy of 95%. In comparison, the Shure SM7B, while stable under the same conditions, has slightly lower accuracy than the optimized SAMSON Meteor Mic and shows a slight decline in performance when the angle exceeds 60°. On the other hand, the Blue Yeti X, although having a wider pickup range, suffers significant performance degradation, particularly when there are large angle changes and strong background noise, with an accuracy of around 92%. The SAMSON Meteor Mic, with precise signal synchronization and enhanced noise suppression techniques, ensures high stability of the system at various angles and distances, making it perform excellently in a variety of application scenarios.

#### 5.3.8. Impact of User Diversity

**Figure 16:**
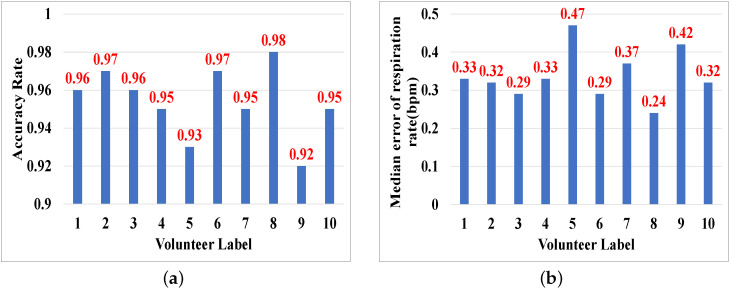
Impact of user diversity. (**a**) Respiration monitoring. (**b**) Cough detection.

To evaluate the impact of user diversity, we display the respiratory rate median errors and cough detection accuracies for all ten volunteers in Figure 16. We can observe from the results that the performance is mainly related to the user’s body size. Specifically, a fatter or thinner body will decrease the sensing performance. For a fatter body, the direction of chest motion is disordered so that the detected displacement of chest motion becomes small. For a thinner body, the small body size leads to a weak chest motion.

#### 5.3.9. Impact of Ambient Noise

**Figure 17:**
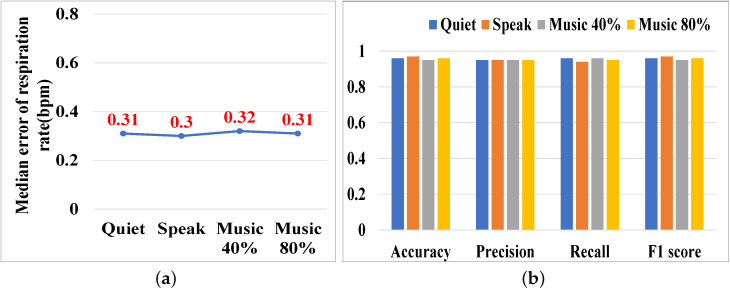
Impact of ambient noise. (**a**) Respiration monitoring. (**b**) Cough detection.

Given that our $P^{3}Warning$ system is based on acoustic signals, we placed a volunteer 2 m away from the transceiver and had the volunteer hold the iPhone playing music behind him to simulate a noise source, i.e., the background noise was approximately 2 m away from the transceiver. The first noise source was human speech, with a volunteer reading an article at a typical volume. The second was music from a mobile phone played at 40% and 80% of its maximum volume. We measured the sound pressure level at the transceiver’s location using the Decibel X app on an iPhone 13 Pro. The decibel levels for the four scenarios were as follows: quiet (35.7 dB), speech (56.6 dB), music at 40% volume (64.9 dB), and music at 80% volume (68.4 dB). Figure 17 shows that the system’s respiration monitoring and cough detection capabilities were consistent across different noise conditions. This is because the ambient noise frequencies were below 14 kHz, which do not interfere with $P^{3}Warning$’s signals in the 18 to 22 kHz frequency band.

### 5.4. Experiments in a Coal Mine Laboratory

#### 5.4.1. Implementation

**Figure 18:**
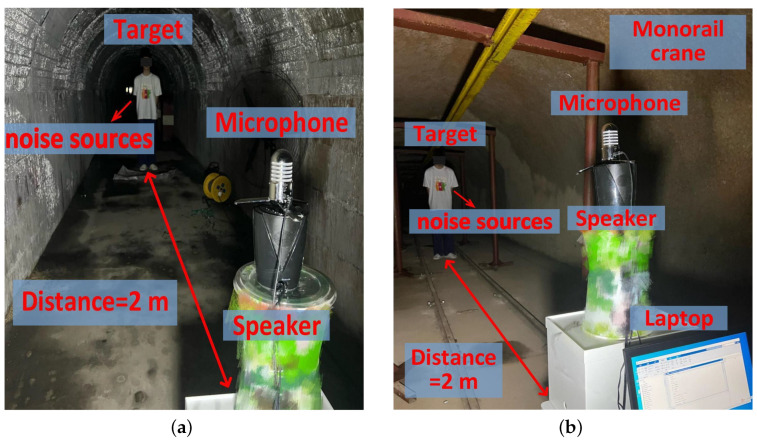
Experiment scenario in a coal mine laboratory. (**a**) Tunnel with some sundries. (**b**) Tunnel with a monorail crane.

We carry out experiments in a coal mine laboratory in our university, which provides an underground coal mine simulation environment. As shown in Figure 18, there are two scenarios in the coal mine laboratory, where Figure 18a shows a straight tunnel with some sundries and Figure 18b shows another tunnel with a monorail crane. The experiment setup is the same as that in the indoor laboratory, with each test conducted by a volunteer, and the distance between the volunteer and the transceiver set to 2 m.

In the two scenarios of the coal mine laboratory, we simulated real mine environment parameters to evaluate the adaptability of the $P^{3}Warning$ system. In Scenario 1 (tunnel with debris), the noise level was relatively low (68.5 dB), air quality was moderate (AQI 110), and dust concentration was 2.5 mg/m^3^. In Scenario 2 (tunnel with a monorail crane), the noise level was higher (76.2 dB), air quality was poorer (AQI 135), and dust concentration reached 4.2 mg/m^3^. The temperature and humidity were 18.5°C/62% and 22.3°C/75%, respectively, where higher humidity and dust levels could potentially affect acoustic signal propagation.

#### 5.4.2. Sensing Performance in the Coal Mine Laboratory

**Figure 19:**
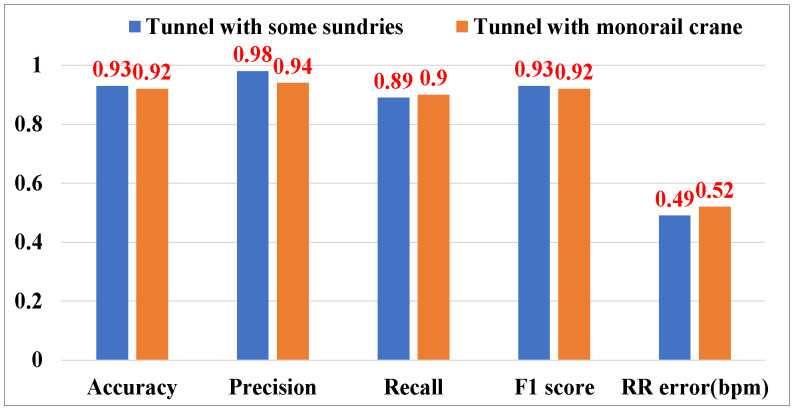
Experimental results in the coal mine laboratory.

As shown in Figure 19, $P^{3}Warning$ can also perform well in the coal mine laboratory. The respiratory rate median errors of two scenarios are 0.49 bpm and 0.52 bpm, respectively. The accuracies of cough detection of two scenarios are 93% and 92%, respectively. Because of a more complex environment, the performance in the coal mine laboratory is slightly lower than that in the indoor laboratory. However, the experimental results demonstrate that $P^{3}Warning$ still works effectively in a complex underground coal mine environment.

## 6. Discussion

### 6.1. Multi-Target Sensing

In this paper, our proposed framework achieves single-target sensing. Although the received signal contains signals reflected from multiple targets, it is challenging to separate each target’s information. When multiple targets are far away from each other and at different angles, we can extract the signal of each individual target by different frequencies. However, if the distance between two targets becomes closer, the sensing performance will be lower. In our future work, we plan to employ beamforming with a microphone array, to separate multiple targets and enhance the signal amplitude.

### 6.2. Motion Interference

Current respiration sensing based on wireless signals (such as acoustic, radar, WiFi, and LoRa) is only effective for static targets. Because the signal variations induced by target activities are much larger than respiration, the respiration-induced signal variations will be submerged and difficult to be extracted. For cough detection, we can rely on the rapidity and abruptness of the cough to distinguish it from many target activities. However, the cough detection performance will still degrade greatly when the target is not static.

### 6.3. Practical Usage

Our proposed $P^{3}Warning$ mainly serves the high-incidence population of pneumoconiosis, such as coal miners. Current pneumoconiosis detection methods are based on lung imaging and pulmonary function tests with professional medical devices. However, there are two problems of these methods: (i) one medical examination costs much money in the hospital, and (ii) since pneumoconiosis is latent, pneumoconiosis is already advanced when a person is diagnosed. $P^{3}Warning$ is based on the early symptoms (i.e., dyspnea, tachypnea, and cough) to detect potential pneumoconiosis. Our proposed method cannot confirm the diagnosis of pneumoconiosis, but alerts the high-incidence population with the possibility of pneumoconiosis. Moreover, $P^{3}Warning$ can be deployed on most commercial audio devices at home and in the work environment.

## 7. Conclusions

This paper describes the $P^{3}Warning$ system, a low-cost, non-contact monitoring and early warning system based on acoustic signals designed for the early detection of pneumoconiosis symptoms using inaudible sound signals. The system is based on commercially available loudspeakers and microphones, and utilizes its non-invasive, highly sensitive, and multi-dimensional information to address a significant challenge in non-invasive health monitoring. Experimental results show that $P^{3}Warning$ performs well in detecting abnormal breathing patterns and coughing, making it particularly suitable for early identification of pneumoconiosis in high-risk groups such as coal miners.

Looking ahead, the system currently supports single-patient monitoring but will be upgraded for multi-target monitoring in the future. This will enhance its versatility in complex environments like workplaces. However, challenges remain in real-world applications, such as environmental factors (e.g., background noise, signal attenuation) affecting acoustic signals and potential accuracy issues in multi-target scenarios. Future research will focus on improving robustness against interference, enhancing multi-target sensing, and optimizing algorithms for more complex applications.

## Data Availability

Dataset available on request from the authors.
